# Worth one’s salt

**DOI:** 10.1007/s12471-018-1190-5

**Published:** 2018-10-18

**Authors:** A. E. Schaafsma, E. A. van der Have, H. Lameijer

**Affiliations:** Department of Emergency Medicine, Medical Centre Leeuwarden, Leeuwarden, The Netherlands

## Answer

The patient showed clinical signs of a combined sedative (shallow breathing, delayed capillary refill time, GCS score E1 M1 V1) and anticholinergic toxidrome (tachycardia, dilated pupils). Therefore, a combined intoxication was suspected. The electrocardiogram (ECG) in the question shows tachycardia with severely broadened QRS duration and QTc interval, with a Brugada-like pattern (right bundle branch block-like pattern with coved ST-segment elevation in V1–3 followed by a negative T wave). This is, among others, observed with sodium channel blockage. An intoxication with at least a tricyclic antidepressant (TCA, which is a sodium channel blocker with anticholinergic effects) was therefore suspected [1, 2].

When a TCA intoxication causes QRS widening, sodium bicarbonate (8.4%) infusion should be started to prevent progression to ventricular dysrhythmias. Sodium bicarbonate is useful not only because of the addition of sodium, but also because of the alkalinisation of the serum and urine, and therefore improved excretion of TCA toxins [1]. Infusion should continue until the QRS normalises [1, 2]. Activated charcoal or forced laxation is only indicated when administrated within 1 h after ingestion or when slow release medication is ingested. When a TCA intoxication leads to the need for cardiopulmonary resuscitation (CPR), administration of most anti-arrhythmic agents (1A and 1C, including amiodarone) should be avoided despite current CPR protocols because of an additional sodium channel blocking effect. Overdrive pacing can be beneficial but difficult, as the sodium channel blockage makes the heart less excitable, which again emphasises the need for sodium bicarbonate therapy. Additionally, administration of magnesium is an option. As a final step, during CPR intra-lipid could be used. However, the latter option is based on expert opinion and case reports [1, 3].

**Fig. 1:**
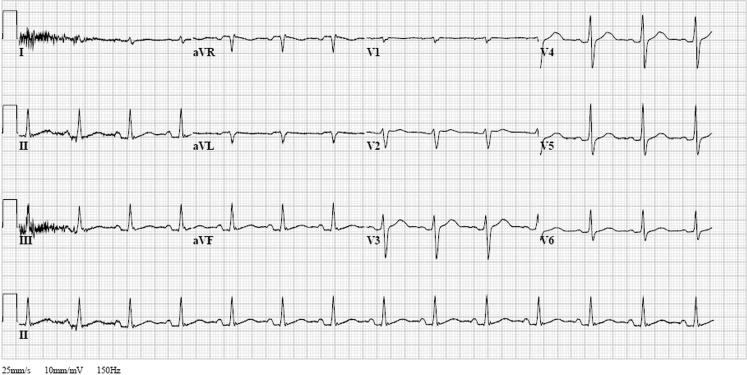
ECG after sodium bicarbonate infusion, with almost complete normalisation

This patient was admitted to the intensive care unit. Soon after sodium bicarbonate infusion the ECG of this patient normalised, as demonstrated in Fig. 1. Laboratory testing showed an intoxication with amitriptyline, among other substances. The patient appeared to be suffering from a psychosis, for which he was admitted to the psychiatric ward after appropriate medical resuscitation.
